# Supplementary figures and images for: Stage-specific control of oligodendrocyte survival and morphogenesis by TDP-43
Source: eLife. 2022 Mar 21;11:e75230. doi: 10.7554/eLife.75230 (PMC8970587; doi:10.7554/eLife.75230)

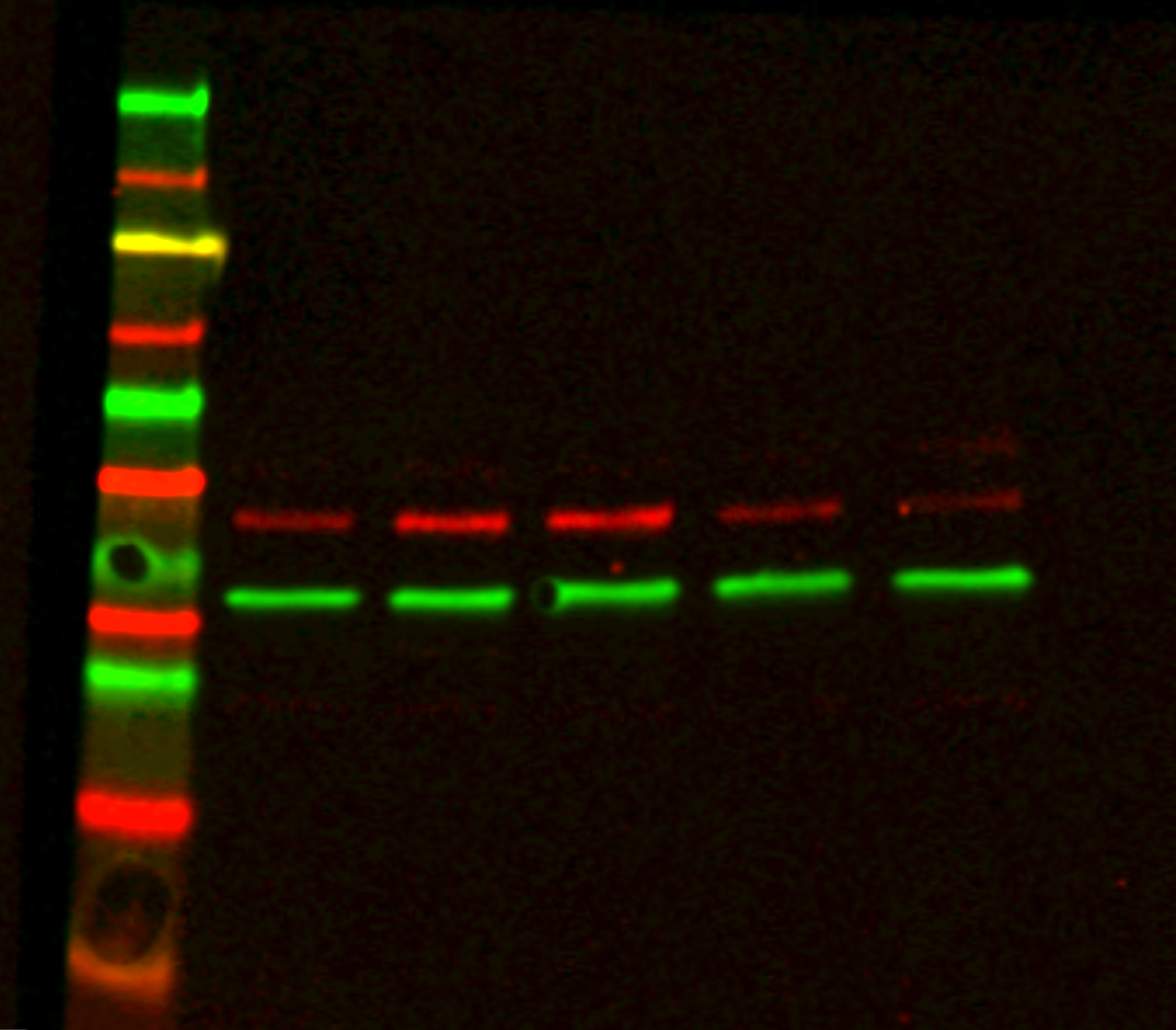

Supplement: Figure 6—source data 1. — The molecular weight of Ermin is 42 kDa and of GAPDH is 37 kDa. [file elife-75230-fig6-data1.zip › 20210413 Ermin-red GAPDH-green.tif]

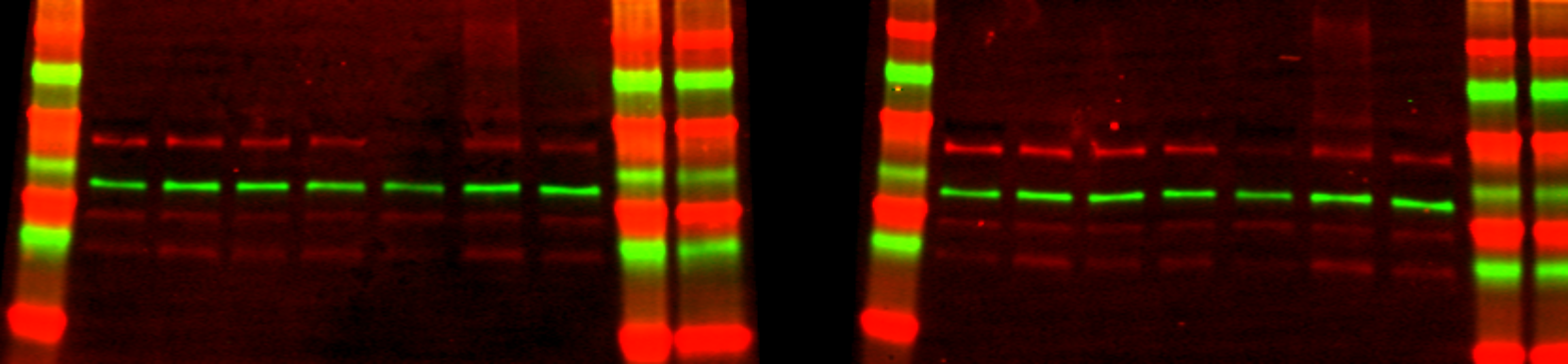

Supplement: Figure 6—source data 1. — The molecular weight of Ermin is 42 kDa and of GAPDH is 37 kDa. [file elife-75230-fig6-data1.zip › 20210521 Ermin-red GAPDH-green.tif]

20210413 Ermin-red GAPDH-green

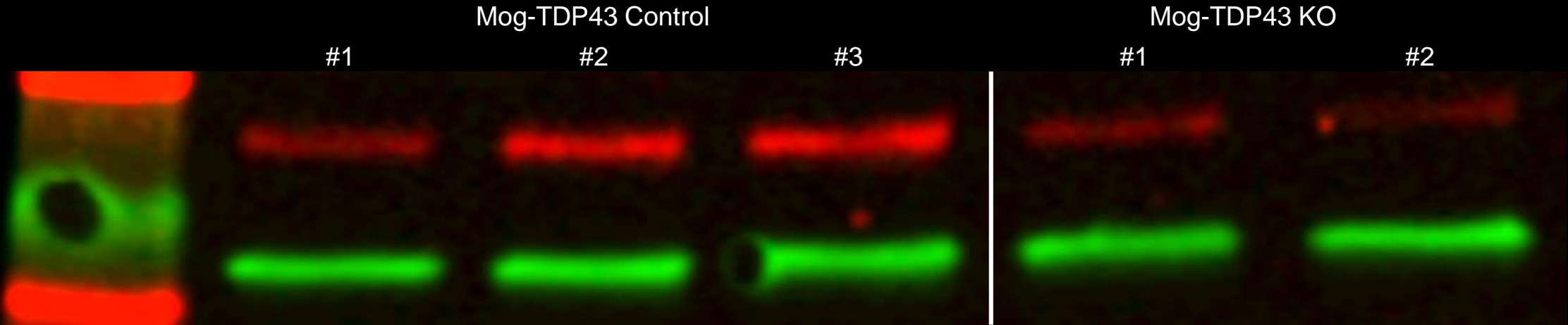

20210521 Ermin-red GAPDH-green

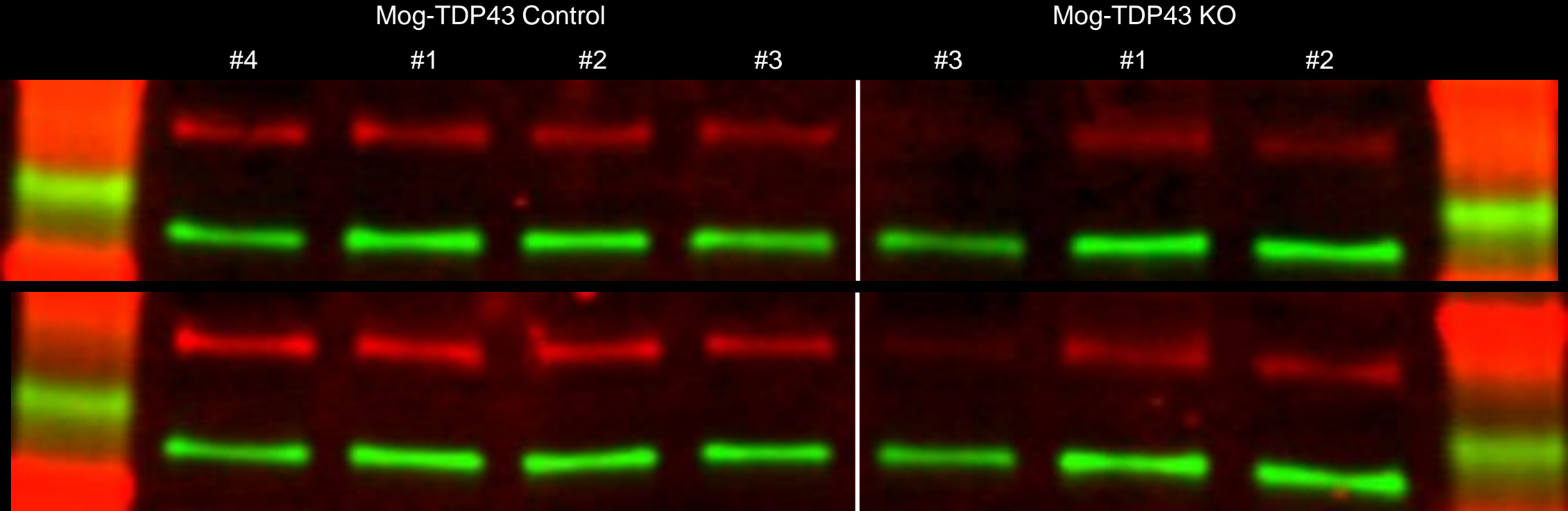

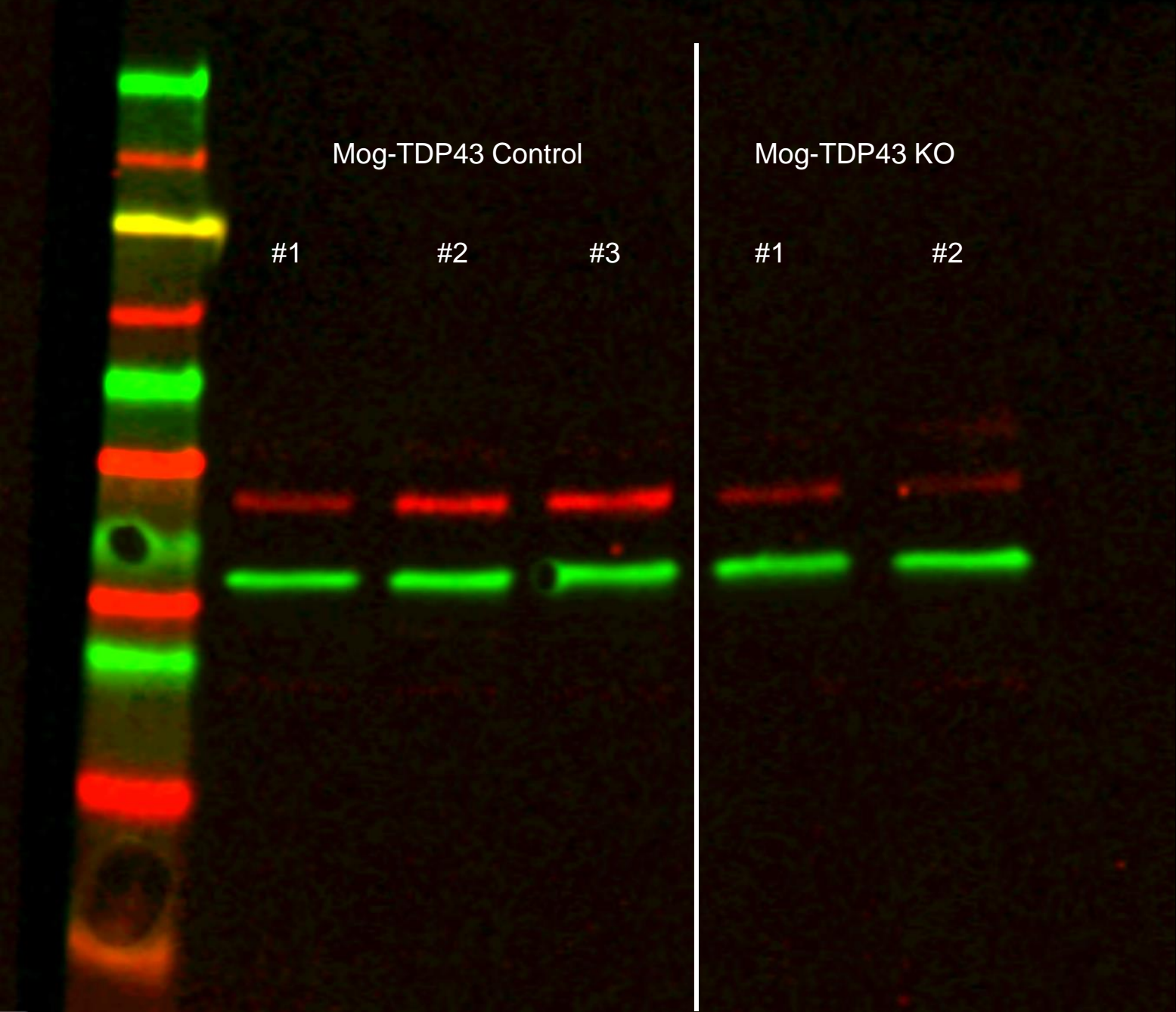

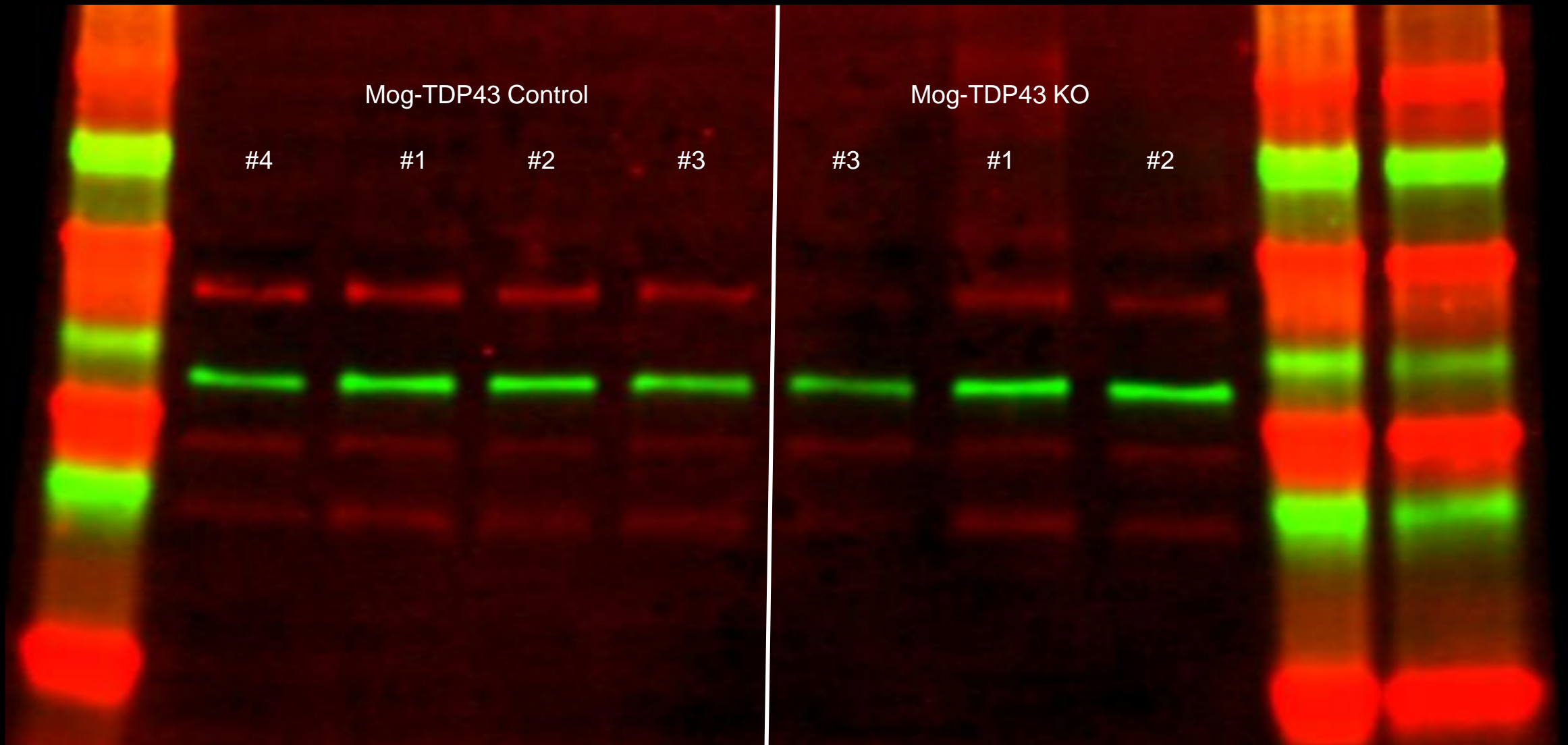

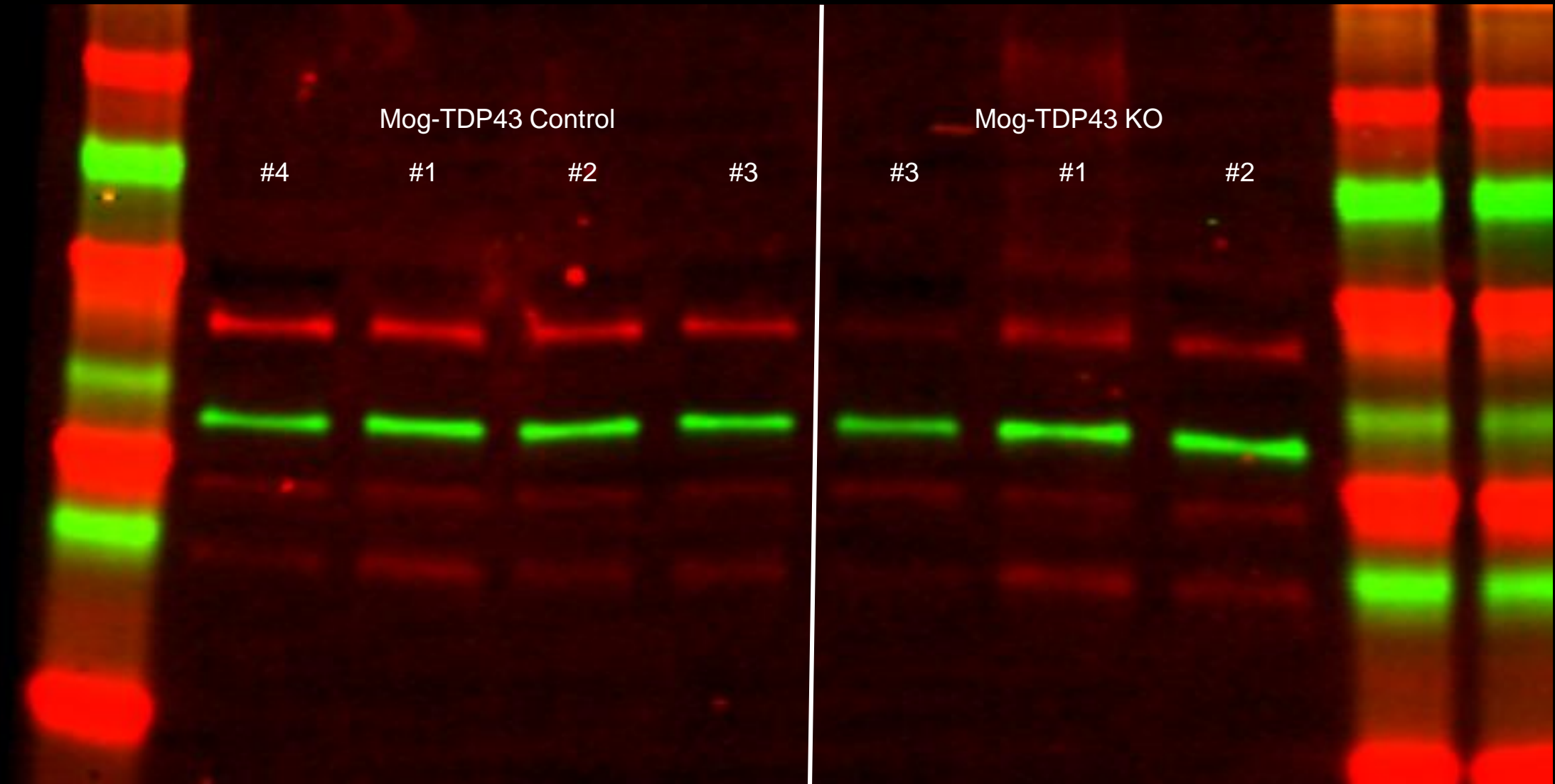

Supplement: Figure 6—source data 1. — The molecular weight of Ermin is 42 kDa and of GAPDH is 37 kDa. [file elife-75230-fig6-data1.zip › Annotated raw and cropped WB images.pdf]
