# Supplementary material for: Stage-specific control of oligodendrocyte survival and morphogenesis by TDP-43
Source: eLife. 2022 Mar 21;11:e75230. doi: 10.7554/eLife.75230 (PMC8970587; doi:10.7554/eLife.75230)
Supplement: Transparent reporting form [file elife-75230-transrepform1.docx]

***eLife’s* transparent reporting form**

We encourage authors to provide detailed information *within their submission* to facilitate the interpretation and replication of experiments. Authors can upload supporting documentation to indicate the use of appropriate reporting guidelines for health-related research (see [EQUATOR Network](http://www.equator-network.org/%20)), life science research (see the [BioSharing Information Resource](https://biosharing.org/)), or the [ARRIVE guidelines](http://www.plosbiology.org/article/info:doi/10.1371/journal.pbio.1000412) for reporting work involving animal research. Where applicable, authors should refer to any relevant reporting standards documents in this form.

If you have any questions, please consult our Journal Policies and/or contact us: [editorial@elifesciences.org](mailto:editorial@elifesciences.org).

**Sample-size estimation**

- You should state whether an appropriate sample size was computed when the study was being designed
- You should state the statistical method of sample size computation and any required assumptions
- If no explicit power analysis was used, you should describe how you decided what sample (replicate) size (number) to use

Please outline where this information can be found within the submission (e.g., sections or figure legends), or explain why this information doesn’t apply to your submission:

Animal care and use under Materials and methods section describes: All experiments were initially performed with a sample size of 3 (biological replicates) for quantification and statistical analyses, and power analysis was then performed to determine the minimum sample size to achieve sufficient statistical power to detect the effect of TDP-43 loss in different transgenic mouse lines at different timepoints.

**Replicates**

- You should report how often each experiment was performed
- You should include a definition of biological versus technical replication
- The data obtained should be provided and sufficient information should be provided to indicate the number of independent biological and/or technical replicates
- If you encountered any outliers, you should describe how these were handled
- Criteria for exclusion/inclusion of data should be clearly stated
- High-throughput sequence data should be uploaded before submission, with a private link for reviewers provided (these are available from both GEO and ArrayExpress)

Please outline where this information can be found within the submission (e.g., sections or figure legends), or explain why this information doesn’t apply to your submission:

Statistical analysis under Materials and methods section describes: All technical replicates (e.g. different brain sections for IHC) for each sample were averaged as a single data point. & n represents the number of animals (biological replicates) used in each experiment.

**Statistical reporting**

- Statistical analysis methods should be described and justified
- Raw data should be presented in figures whenever informative to do so (typically when N per group is less than 10)
- For each experiment, you should identify the statistical tests used, exact values of N, definitions of center, methods of multiple test correction, and dispersion and precision measures (e.g., mean, median, SD, SEM, confidence intervals; and, for the major substantive results, a measure of effect size (e.g., Pearson's r, Cohen's d)
- Report exact p-values wherever possible alongside the summary statistics and 95% confidence intervals. These should be reported for all key questions and not only when the p-value is less than 0.05.

Please outline where this information can be found within the submission (e.g., sections or figure legends), or explain why this information doesn’t apply to your submission:

Statistical analysis under Materials and methods section describes: Significance was determined using one-way ANOVA test with Tukey’s correction for multiple comparisons or unpaired, two-tailed Student’s t-test. Each figure legend otherwise contains the statistical tests used to measure significance and the corresponding significance level (p-value). n represents the number of animals (biological replicates) used in each experiment. Data are reported as mean ± SEM and p-value < 0.05 was considered statistically significant.

Each datapoint was plotted on the bar graphs whenever possible.

No data were excluded from the analyses.

(For large datasets, or papers with a very large number of statistical tests, you may upload a single table file with tests, Ns, etc., with reference to sections in the manuscript.)

**Group allocation**

- Indicate how samples were allocated into experimental groups (in the case of clinical studies, please specify allocation to treatment method); if randomization was used, please also state if restricted randomization was applied
- Indicate if masking was used during group allocation, data collection and/or data analysis

Please outline where this information can be found within the submission (e.g., sections or figure legends), or explain why this information doesn’t apply to your submission:

This is not relevant as different experimental groups were defined by the animals' genotypes (Control vs. KO or cKO).

Image processing and analysis under Materials and methods section describes: Density and g-ratio quantifications were performed by a blinded observer.

**Additional data files (“source data”)**

- We encourage you to upload relevant additional data files, such as numerical data that are represented as a graph in a figure, or as a summary table
- Where provided, these should be in the most useful format, and they can be uploaded as “Source data” files linked to a main figure or table
- Include model definition files including the full list of parameters used
- Include code used for data analysis (e.g., R, MatLab)
- Avoid stating that data files are “available upon request”

Please indicate the figures or tables for which source data files have been provided:

Supplementary Data – Differential gene expression Excel file of bulk RNA-Seq.xlsx is provided for Figure 6B, Figure 6—figure supplement 1, Figure 6—figure supplement 2, and Figure 6—figure supplement 4.
